# Supplementary material for: Comparison of Multiple Strategies for Precision Transgene Knock-In in Gallus gallus Genome via Microhomology-Mediated End Joining
Source: Int J Mol Sci. 2023 Oct 29;24(21):15731. doi: 10.3390/ijms242115731 (PMC10649300; doi:10.3390/ijms242115731)
Supplement: Supplementary file 1 [file ijms-24-15731-s001.zip › Supplementary Figure S1.pdf]

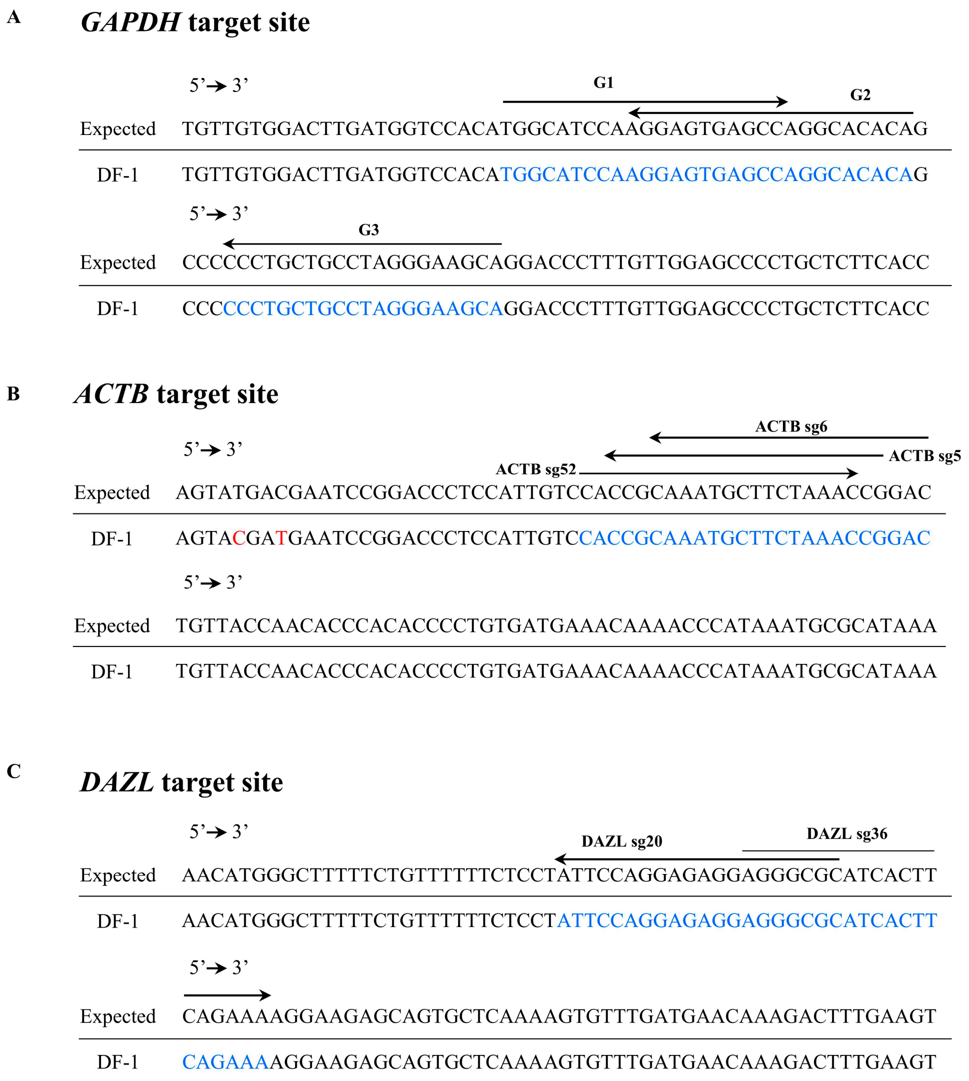

Figure S1. Sequence analysis of sgRNA target sites in DF-1 cells.

(A-C) Arrow lines indicate the sgRNA. Red letters indicate mutations between the sequence analysis and the expected sequence from NCBI.
