# Supplementary material for: Comparison of Multiple Strategies for Precision Transgene Knock-In in Gallus gallus Genome via Microhomology-Mediated End Joining
Source: Int J Mol Sci. 2023 Oct 29;24(21):15731. doi: 10.3390/ijms242115731 (PMC10649300; doi:10.3390/ijms242115731)
Supplement: Supplementary file 1 [file ijms-24-15731-s001.zip › Supplementary Tables S1-S3.pdf]

Comparison of multiple strategies for precision transgene knock-in in *Gallus gallus* Genome via microhomology-mediated end joining

Table S1. sgRNA sequences used for Cas9 plasmid

sgRNAs	Sequence (5'->3')
GAPDH G1-F	CACCG TGGCATCCAAGGAGTGAGCC
GAPDH G1-R	AAAC GGCTCACTCCTTGGATGCCA C
GAPDH G2-F	CACCG TGTGTGCCTGGCTCACTCCT
GAPDH G2-R	AAAC AGGAGTGAGCCAGGCACACA C
GAPDH G3-F	CACCG TGCTTCCCTAGGCAGCAGGG
GAPDH G3-R	AAAC CCCTGCTGCCTAGGGAAGCA C
ACTB A5-F	CACCG CGGTTTAGAAGCATTTGCGG
ACTB A5-R	AAAC CCGCAAATGCTTCTAAACCG C
ACTB A6-F	CACCG GTCCGGTTTAGAAGCATTTG
ACTB A6-R	AAAC CAAATGCTTCTAAACCGGAC C
ACTB A52-F	CACCG CACCGCAAATGCTTCTAAAC
ACTB A52-R	AAAC GTTTAGAAGCATTTGCGGTG C
DAZL D20-F	CACCGGCGCCCTCCTCTCCTGGAAT
DAZL D20-R	AAACATTCCAGGAGAGGAGGGCGCC
DAZL D36-F	CACCGAGGGCGCATCACTTCAGAAA
DAZL D36-R	AAACTTTCTGAAGTGATGCGCCCTC

F: forward primer; R: reverse primer.

Table S2. Primers used for genotyping and sequencing

Primers	Sequence (5'->3')
GAPDH-3F	GGTACTTCTGGTTCAGGCTGTTAT
GAPDF-3R	GTTGTCAGTAGAGCAGCAGGTATG
GAPDH-4F	CTCTAGTGAGATGGCTTCGTATCTG
GAPDH-4R	GGAGACTGCTCAGGAAACATAAGG
ACTB-1F	GATGGAAACTGCTTTGTCTTAGTG
ACTB-1R	CTCAGATGCATTGTAGAACTTTGG
DAZL-1F	GTTAGGTCAGTACCTGGTCATC
DAZL-1R	GTTACACCGTGACGTCTTCT

DAZL-2F	CTTGTAAACGAGAGAGCAGAGTTA
DAZL-2R	CTGCAAGAGGTAACTGGTTGA

F: forward primer; R: reverse primer.

Table S3. Primers used for the EGFP donor vector

Primers	Sequence (5'->3')
G1-20F-EGFP Oligo1	GCCAGGCACACAGCCCCC
G1-20F-EGFP Oligo2	GCACTCCTTGGATGCCATGTGC
G1-20F-EGFP Oligo3	GCATCCAAGGAGTGCCCGGATCCGGCTCCGGTGC
G1-20F-EGFP Oligo4	GGCTGTGTGCCTGGCTCAGGCACCGGGCTTGCG
G1-20R-EGFP Oligo1	GCCAGGCACACAGCCCCC
G1-20R-EGFP Oligo2	GCACTCCTTGGATGCCATGTGC
G1-20R-EGFP Oligo3	ACATGGCATCCAAGGAGTGCCCATAGAGCCCACCGCATC
G1-20R-EGFP Oligo4	CGGTGCCTGATCTAGAGGGCCCGTTTAAACCC
G1-20R-EGFP Oligo5	GCCCTCTAGATCAGGCACCGGGCTTGCG
G1-20R-EGFP Oligo6	AGGGGGGCTGTGTGCCTGGCCCGGATCCGGCTCCGGTGC
G1-40F-EGFP Oligo1	GCCAGGCACACAGCCCCC
G1-40F-EGFP Oligo2	GCACTCCTTGGATGCCATGTGG
G1-40F-EGFP Oligo3	GCATCCAAGGAGTGCCCGGATCCGGCTCCGGTGC
G1-40F-EGFP Oligo4	GGCTGTGTGCCTGGCTCAGGCACCGGGCTTGCG
G1-40R-EGFP Oligo1	GCCAGGCACACAGCCCCC
G1-40R-EGFP Oligo2	GCACTCCTTGGATGCCATGTGG
G1-40R-EGFP Oligo3	GCATCCAAGGAGTGCCCGGATCCGGCTCCGGTGC
G1-40R-EGFP Oligo4	GGCTGTGTGCCTGGCTCAGGCACCGGGCTTGCG
G2-20R-EGFP Oligo1	ACGCGTACGTGTTTGGTGGTCCACATGGCATCCAA-GCCCCCATAGAGCCCACCGCATCCCC
G2-20R-EGFP Oligo2	CAAACACGTACGCGTACGATGCTATGTAACGC
G2-20R-EGFP Oligo3	TCTAGAATGCTGATGGGCTAGCAAAATCAGCCTC

G2-20R-EGFP Oligo4	CATCAGCATTCTAGAGCATCGTACGCGTACGTGTTT-GGGGGGCTGTGTGCCTGGCTCACCGGATCCGGCTCCGGTG
G3-20R-EGFP Oligo1	ACGCGTACGTGTTTGG-GAGCCAGGCACACAGCCCCCCCCCATAGAGCCCACCG-CATCCCC
G3-20R-EGFP Oligo2	CAAACACGTACGCGTACGATGCTATGTAACGC
G3-20R-EGFP Oligo3	TCTAGAATGCTGATGGGCTAGCAAAATCAGCCTC
G3-20R-EGFP Oligo4	CATCAGCATTCTAGAGCATCGTACGCGTACGTGTTT-GGGGTCCTGCTTCCCTAGGCAGCCGGATCCGGCTCCGGTG
A6-20R-EGFP Oligo1	CCGCGTTACATAGCATCGTACGCGTACGTGTTT-GGCCCTCCATTGTCCACCGCAACCCATAGAGCCCACCG-CATCCC
A6-20R-EGFP Oligo2	ACGCGTACGTGTTTGGACAGTCCGGTTTAGAAGCATCCG-GATCCGGCTCCGGTG
A6-20R-EGFP Oligo3	TGCTATGTAACGCGGAACTCCATATATGGG
A6-20R-EGFP Oligo4	CAAACACGTACGCGTACGATGCTCTAGAATG
D36-20R-EGFP Oligo1	CCGCGTTACATAGCATCGTACGCGTACGTGTTTGGAG-GAGGGCGCATCACTTCAGCCCATAGAGCCCACCGCATCCC
D36-20R-EGFP Oligo2	ACGCGTACGTGTTTGGTGAGCACTGCTCTTCCTTTTCCG-GATCCGGCTCCGGTG
D36-20R-EGFP Oligo3	TGCTATGTAACGCGGAACTCCATATATGGG
D36-20R-EGFP Oligo4	CAAACACGTACGCGTACGATGCTCTAGAATG
